# The sustained PGE_2_ release matrix improves neovascularization and skeletal muscle regeneration in a hindlimb ischemia model

**DOI:** 10.1186/s12951-022-01301-3

**Published:** 2022-02-24

**Authors:** Haoyan Huang, Shang Chen, Hui Cheng, Jiasong Cao, Wei Du, Jun Zhang, Yuqiao Chang, Xiaohong Shen, Zhikun Guo, Zhibo Han, Guoqiang Hua, Zhong-Chao Han, Nadia Benkirane-Jessel, Ying Chang, Zongjin Li

**Affiliations:** 1 Nankai University School of Medicine, Tianjin, China; 2 The Key Laboratory of Bioactive Materials, Ministry of Education, The College of Life Sciences, Nankai University, Tianjin, China; 3 Tianjin Key Laboratory of Human Development and Reproductive Regulation, Tianjin Central Hospital of Gynecology Obstetrics, Nankai University Affiliated Hospital of Obstetrics and Gynecology, Tianjin, China; 4 Tianjin Key Laboratory of Blood Cell Therapy Technology, Union Stem Cell & Gene Engineering Co. Ltd., Tianjin, China; 5 Department of Pain Treatment, Tianjin Union Medical Center, Nankai University Affiliated Hospital, Tianjin, China; 6 Henan Key Laboratory of Medical Tissue Regeneration, Xinxiang Medical University, Xinxiang, China; 7 Tianjin Key Laboratory of Engineering Technologies for Cell Pharmaceutical, National Engineering Research Center for Cell Products, AmCellGene Co., Ltd., Tianjin, China; 8 Beijing Engineering Laboratory of Perinatal Stem Cells, Beijing Institute of Health and Stem Cells, Health & Biotech Co., Beijing, China; 9 INSERM (French Institute of Health and Medical Research), UMR 1260, Regenerative Nanomedicine (RNM), FMTS, Strasbourg, France; 10 Faculté de Chirurgie Dentaire, Université de Strasbourg, Strasbourg, France; 11 State Key Laboratory of Kidney Diseases, Chinese PLA General Hospital, Beijing, China

**Keywords:** Prostaglandin E_2_ (PGE_2_), Sustained release, Hindlimb ischemia (HI), Angiogenesis, Muscle regeneration, MyoD1, Molecular imaging

## Abstract

**Background:**

The promising therapeutic strategy for the treatment of peripheral artery disease (PAD) is to restore blood supply and promote regeneration of skeletal muscle regeneration. Increasing evidence revealed that prostaglandin E_2_ (PGE_2_), a lipid signaling molecule, has significant therapeutic potential for tissue repair and regeneration. Though PGE_2_ has been well reported in tissue regeneration, the application of PGE_2_ is hampered by its short half-life in vivo and the lack of a viable system for sustained release of PGE_2_.

**Results:**

In this study, we designed and synthesized a new PGE_2_ release matrix by chemically bonding PGE_2_ to collagen. Our results revealed that the PGE_2_ matrix effectively extends the half-life of PGE_2_ in vitro and in vivo. Moreover, the PGE_2_ matrix markedly improved neovascularization by increasing angiogenesis, as confirmed by bioluminescence imaging (BLI). Furthermore, the PGE_2_ matrix exhibits superior therapeutic efficacy in the hindlimb ischemia model through the activation of MyoD1-mediated muscle stem cells, which is consistent with accelerated structural recovery of skeletal muscle, as evidenced by histological analysis.

**Conclusions:**

Our findings highlight the chemical bonding strategy of chemical bonding PGE_2_ to collagen for sustained release and may facilitate the development of PGE_2_-based therapies to significantly improve tissue regeneration.

**Graphical Abstract:**

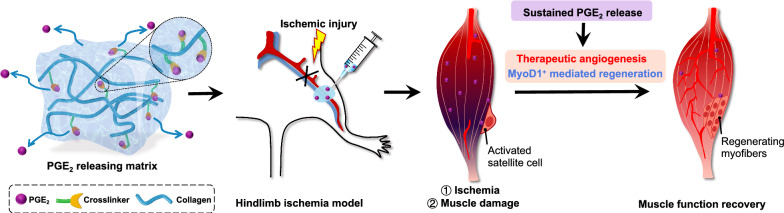

**Supplementary Information:**

The online version contains supplementary material available at 10.1186/s12951-022-01301-3.

## Introduction

With an increase in the elderly population, the prevalence and incidence of peripheral artery disease (PAD) are increasing markedly and globally [1]. PAD as a degenerative vascular disease affects mainly blood flow and further causes critical limb ischemia (CLI), which portends a high rate of amputation along with patient mortality [2–4]. Interrupting the blood supply to the ischemic limb further hinders the delivery of adequate nutrients and oxygen, leading to skeletal muscle damage [5]. Standard therapies, which are surgery and endovascular revascularization, are not particularly effective and can only offer a cure for ~ 20–40% of patients, indicating the need to develop new therapeutic strategies for the treatment of PAD [4, 6].

The overarching therapeutic strategy for PAD treatment is the restoration of blood supply via new vessels in ischemic tissue so that the cellular response can occur normally in the ischemic area [7]. In addition to recovery of blood supply, regeneration of skeletal muscle is a crucial therapeutic target that must be considered [8]. When skeletal muscle is damaged by stimuli such as ischemia, resident muscle stem cells, also termed satellite cells (SCs), become activated and immediately participate in regenerative processes through cell proliferation and differentiation to form new muscle fibers [6, 9]. Myogenic regulatory factors such as MyoD1 (myoblast determination protein 1) and Myf5 (myogenic factor 5) are specific markers that promote SC differentiation into myotubes and play a major role in the regulation of skeletal muscle regeneration during myogenesis [10, 11].

Prostaglandin E_2_ (PGE_2_) is a very potent lipid mediator and is involved in several biological processes, including inflammation, angiogenesis, blood pressure, and fertility [12–14]. PGE_2_ is synthesized by cyclooxygenase (COX) and prostaglandin E synthases (PGE_S_) from arachidonic acid and binds to G protein-coupled receptors (GPCRs), including EP1, EP2, EP3 and EP4, and further causing various downstream effects [13]. PGE_2_ is considered a promising candidate molecule to improve tissue repair and regeneration in ischemic disease [13, 15]. The available evidence suggests that PGE_2_ could ameliorate tissue ischemia by stimulating angiogenesis, improving both vascular densities, and promoting the secretion of pro-angiogenic cytokines [16–18]. In addition, the COX-2/PGE_2_ pathway is essential during the early stages of skeletal muscle regeneration by promoting stem cell myogenic differentiation and regulating the immune response [19–21]. However, under physiological conditions, PGE_2_ has a half-life of approximately 2.5 to 5 min [22], which limits its applications in regenerative therapy. Therefore, promising controlled release strategies need to be developed for long-term release PGE_2_ [13, 15].

Collagen, the most abundant protein component of the extracellular matrix (ECM), is a major component in a wide range of drug delivery systems and biomaterial applications due to its excellent properties, including weak antigenicity, good permeability, biocompatibility, biodegradability, and collagen-based matrix could closely mimic the native tissue microenvironment [23, 24]. More importantly, purified collagen is chemically defined and provides functional groups for chemical modification to obtain desired results, such as sustained release of therapeutic molecules [25]. Therefore, we designed and synthesized a new PGE_2_-releasing collagen matrix, denoted as the PGE_2_ matrix, chemically bonding PGE_2_ to collagen by cross-linking hydrazone. This PGE_2_ delivery system has a slow-release property under physiological conditions, which is an applicable solution to the short clearance time of PGE_2_ in the circulation. We hypothesized that this PGE_2_ matrix could effectively extend the half-life of PGE_2_ and exhibit superior therapeutic efficacy in a mouse model of hindlimb ischemia (HI). We evaluated the role of the PGE_2_ matrix on pro-angiogenesis in vitro and in vivo. The pro-angiogenic effects of the PGE_2_ matrix were noninvasively tracked by bioluminescence imaging (BLI). In addition, we further investigated the effect of the PGE_2_ matrix on the promotion of muscle regeneration.

## Methods

### Preparation of the PGE_2_ matrix

In general, PGE_2_ was chemically cross-linked to collagen through the cross-linker (HBA-PEI) to obtain the collagen matrix that releases PGE_2_ (PGE_2_ matrix). In detail, branched polyethylenimine (PEI, Mw = 10,000) of 60 mg and 4-hydroxybenzoic acid (HBA) of 14.8 mg were dissolved in 5 mL of dimethyl sulfoxide (DMSO), and then added hydrochloride of 1-(3-dimethylaminopropyl)-3-ethylcarbodiimide (EDC) of 54 mg and *N*-hydroxy-succinimide (sulfo-NHS) of 32 mg. The reaction proceeded at 37 °C for 24 h under nitrogen atmosphere. After the reaction was stopped, the products (HBA-PEI crosslinker) were dialyzed against deionized water and finally lyophilized [26]. Subsequently, PGE_2_ (CAS 363-24-6; Santa Cruz Biotechnology) of 10 mg and the HBA-PEI crosslinker of 30 mg were dissolved separately in 4 mL of ethanol, then mixed and followed by the addition of 1 mL of glacial acetic acid. The reaction mixture was heated to 80 °C and stirred for 2 h and refluxed by adding molecular sieves under nitrogen for 20 h and allowed to cool at 0 °C. The products (PGE_2_-crosslinker) were dialyzed against deionized water and finally lyophilized. Finally, we link the PGE_2_-cross-linker conjugates to collagen (Collagen I, Rat Tail, Corning) [27]. To activate collagen, 5 mg of EDC and 5 mg sulfo-NHS were added to 3 mL of collagen solution, collected with 2-(*N*-morpholino) ethanesulfonic acid (MES buffer, 50 mM, 4 mL) and reacted for 20 min at room temperature, and then 1.4 μL of 2-mercaptoethanol (final concentration 20 mM) to quench EDC. Separated activated collagen from excess reducing agent and inactivated crosslinker using Ultra-4 centrifugal filter units (100 kDa), and rinsed collagen with 3 mL of MES buffer (pH 7.0, 50 mM). The activated collagen was mixed with PGE_2_ cross-linker conjugates to react overnight with a stir bar at 4 °C and then ultrafiltered and washed with sterile water to remove unreacted conjugates and cross-linking reagents using Ultra-4 centrifugal filter units (100 kDa) at 4 °C. Collagen was collected with PBS and then lyophilized to obtain the product (PGE_2_ matrix).

### Characterization of the PGE_2_ matrix

The rheological measurement of the PGE_2_ matrix was performed using a rheometer (TA Instruments, USA) with a 25 mm parallel plate geometry [28]. The heating temperature range was selected from 2 °C to 44 °C and the heating rate was set to 2 °C/min to measure the rheological properties of the PGE_2_ matrix. The storage modulus (G′) modulus and the loss modulus (G′′) were monitored at a frequency of 1 rad/s. The gelation temperature of the PGE_2_ matrix was characterized by the point when the G′ was equal to G″ and then predominated over G″. After the PGE_2_ matrix was formed, frequency and strain sweep tests were performed to characterize the viscoelastic mechanical properties of the PGE_2_ matrix. For the frequency sweep assay, oscillating shear strains of 1% were applied to the samples with frequencies ranging from 0.05 to 5 Hz. For the strain sweep assay, oscillating shear with strains ranging from 0.1 to 10% was applied to the samples at 1 Hz. Furthermore, scanning electron microscopy (SEM) (FEI, Czech Republic) was used to analyze the morphology of the PGE_2_ matrix after lyophilization. The samples were gold coated before SEM and the accelerating voltage was 10 kV.

### Measurement of PGE_2_ release

For the in vitro PGE_2_ release assay, 50 μL the PGE_2_ matrix with different concentrations (2 μM, 5 μM, and 10 μM) were loaded into a 1.5 mL microcentrifuge and incubated at 37 °C for 15 min to form a gel and then carefully covered with 1 mL of PBS on top of the matrix. Meanwhile, 50 μL the 2 μM PGE_2_ matrix was loaded into a 1.5 mL microcentrifuge and incubated at 37 °C for 15 min to form a gel and then carefully covered with 1 mL of PBS with different pH (7.4 and 6.8) on top of the matrix. All samples were monitored for up to 16 days at 37 °C. At different time points, 50 μL of solution was removed from the supernatant layer and replenished with fresh PBS. The concentration of PGE_2_ was detected by a chemiluminescence enzyme immunoassay (K051-H1, Assay Design, Inc., USA). The PGE_2_ group (PGE_2_ physically mixed with collagen) served as a control to compare the PGE_2_-releasing profile of cross-linked versus unlinked PGE_2_.

For the in vivo PGE_2_ release assay, the PGE_2_ matrix was injected into the ischemic muscle of C57BL/6 albino mice (8–10 weeks old, male, weight 25–30 g, n = 3) at total volume of 75 µL (2 μM) after injury. Mice were anesthetized with avertin at the indicated time points (day1, 3, 7, 10, 14) and transcardially perfused with a PBS solution to prepare muscle tissue homogenates. The tissue samples were homogenized on ice in saline (10% tissue homogenate) with the antioxidant butylated hydroxytoluene (BHT; 10 μM) and the COX inhibitor indomethacin (1 μM) which could block ex vivo arachidonic acid autooxidation and PGs formation. The homogenates were then centrifuged at 14,500 rpm for 45 min. Supernatants were collected to detect PGE_2_ release. PGE_2_ was measured by a chemiluminescence enzyme immunoassay (K051-H1, Assay Design, Inc., USA). The PGE_2_ group (PGE_2_ physically mixed with collagen) served as a control to compare the PGE_2_-releasing profile of cross-linked versus unlinked PGE_2_. The control group (hindlimb ischemia mice injected with collagen) served as a control to reflect the level of PGE_2_ after injury stimulation. The Sham group served as a blank to reflect the baseline level of PGE_2_.

### Mouse model of hindlimb ischemia

In this study, the expression of the vascular endothelial growth factor receptor 2 (VEGFR2) in VEGFR2-Fluc-KI transgenic mice (C57BL/6 albino and outbred (Nu/Nu) background, Xenogen Corp, Hopkinton, USA) could drive the expression of firefly luciferase (Fluc) [29], and was used to monitor angiogenesis in real time in living animals. The treatment of animals and the experimental procedures of the present study adhere to the Nankai University Animal Care and Use Committee Guidelines, which were in line with the animal care guidelines approved by the National Institutes of Health. In summary, we first anesthetized mice (8–10 weeks old, male, weight 25–30 g) with 2.5% avertin intraperitoneal injection (240 mg/kg, Sigma-Aldrich). The femoral artery was ligated after being separated from the femoral vein and nerve, and the upper end of the artery bifurcation near the knee was also ligated. Excised the main branch of the femoral artery between the double nodes to establish the mouse hindlimb ischemia model as previously described [30, 31]. Post-ischemia, a 75 μL total volume of the PGE_2_ matrix was injected into 3 sites (25 μL per site) of the adductor muscle and gastrocnemius muscles, where are the primary ischemic tissues after ligation [32].

### Assessment of limb collateral vessel and limb function

To investigate collateral vessel development, we evaluated microvessel density in the ischemic hindlimbs on day 21 to measure limb angiogenesis after PGE_2_ matrix therapy by angiography. Barium sulfate contrast agent (0.3 g/mL, 10 mL) was infused by transcardiac perfusion. The images were captured using the SPECT/CT imaging System (Mediso, USA) for quantitative angiographic analysis. In addition, semi-quantitative functional evaluations of ischemic limbs were examined in a blinded fashion as previously reported [33].

#### Bioluminescence imaging (BLI)

To real-time track the proangiogenic effects of the PGE_2_ matrix in vivo, VEGFR2-Fluc-KI transgenic mice visualized by bioluminescence imaging (BLI) using the IVIS Lumina II system (Xenogen Corporation, Hopkinton, MA) as previously reported [29, 34]. Anesthetized mice were injected intraperitoneally with D-luciferin (150 mg/kg; Biosynth International, USA) and the expression of VEGFR2 was visualized by BLI [35].

#### Scratch wound healing assay

The scratch wound healing assay was implemented to evaluate the migration capacity of human umbilical vein endothelial cells (HUVECs), which were obtained from the American Type Culture Collection (ATCC, USA) and cultured in EGM2 medium (Lonza, USA). HUVECs were seeded in a 6-well plate coated with PGE_2_ matrix (2 μM), when confluence was reached, scratch wounds were generated using the tip of a 10 μL micropipette. Images of five fields of view were taken at 0 and 12 h. The migratory effect was quantitated using ImageJ software.

### Tube formation assay

The tube formation assay was adopted on a 48-well plate coated with 150 μL Matrigel (Corning, USA) per well [34] to detect the proangiogenic effect of the PGE_2_ matrix. The Matrigel coated plate was placed on ice for 30 min and then placed in an incubator at 37 °C for gelatinization. The PGE_2_ matrix (2 μM) was added to the Matrigel precoated-plate and 3 × 10^4^ HUVECs per well were seeded in Matrigel coated plates. After incubation for 12 h, images in three random fields and the total length of the network structures were measured using ImageJ software as previously described before [34].

### Histology analysis

On days 7, 14, and 28 after injury, mice were sacrificed and damaged muscle samples were harvested. Hematoxylin–Eosin (H&E) and Masson’s Trichrome staining were performed to detect fibrosis of injured limbs. Immunostaining was performed to determine the therapeutic effects of the PGE_2_ matrix. For immunostaining, primary antibodies were used as follow, mouse anti-mouse α-SMA (1:200, BD, USA), mouse anti-mouse Ki-67 (1:200, Invitrogen, USA), rabbit anti-mouse cleaved caspase-3 (1:100, Wanleibio, China) and rat anti-mouse F4/80 (1:100, Abcam, USA), anti-MyoD1 (1:1000, Wanleibio, China), rat anti-mouse CD45 (1:200, BD, USA). Secondary antibodies, Alexa Fluor 594 labeled goat anti-rat, Alexa Fluor 555 labeled goat anti-mouse, Alexa Fluor 488 labeled goat anti-mouse and Alexa Fluor 488 labeled goat anti-rabbit IgG were used. Cell nuclei were counterstained with 4′,6-diamidino-2-phenylindole (DAPI). Immunohistochemistry was developed using a DAB peroxidase substrate DAB staining kit (ZSGB-BIO, China) according to the manufacturer’s instructions. The images were analyzed using ImageJ software.

### Quantitative real-time PCR

Total RNA was extracted using TRIZOL reagent (Takara, Japan) according to the manufacturer’s protocol. RNA was reverse transcribed into the first strand of cDNA by reverse transcriptase (YEASEN, China) using oligodT primers. Subsequently, the mRNA expression levels were quantified using SYBR Green Supermix (YEASEN, China). Real-time PCR analysis was performed on a CFX96 TM Real-Time PCR System (Bio-Rad, USA). The 2^−ΔΔCt^ method was used to analyze relative gene expression. The sequences of the primers are listed in Additional file 1: Tables S1 and S2.

### Western blot analysis

RIPA buffer (Solarbio, China) supplemented with protease inhibitor (Roche, Germany) was used to homogenize and lyse muscle tissue samples to obtain total protein extracts. Protein concentration of protein extracts was measured using a BCA Protein Quantification Kit (Meilunbio, China) according to the manufacturer’s guidelines. The total of 30 µg protein of each sample was run on 10% SDS-PAGE gels in electrophoresis buffer and transferred to nitrocellulose membranes (Millipore, USA) for blotting. Immunoblots were blocked for 2 h at room temperature and incubated overnight at 4 °C with primary antibodies with the following antibodies: rabbit anti-MyoD1 (1:1000, Wanleibio, China) and mouse anti-tubulin (1: 1000, Poteintech, USA). Goat anti-rabbit IgG-HRP (CWBIO, China) and goat anti-mouse IgG-HRP (Beyotime, China) were used as secondary antibodies and protein signals were detected using enhanced chemiluminescence (ECL) (Millipore, USA).

### Statistical analysis

Data were analyzed by one-way ANOVA and subjected to Levene’s test before ANOVA to check. homogeneity of variance. Differences among the means were analyzed by Duncan's multiple-range test using SPSS Statistics, version 25.0 (IBM. Inc. USA). Differences were considered significant at *P* values < 0.05.

## Results

### Establishment of the PGE_2_ slow-release system

**Fig. 1:**
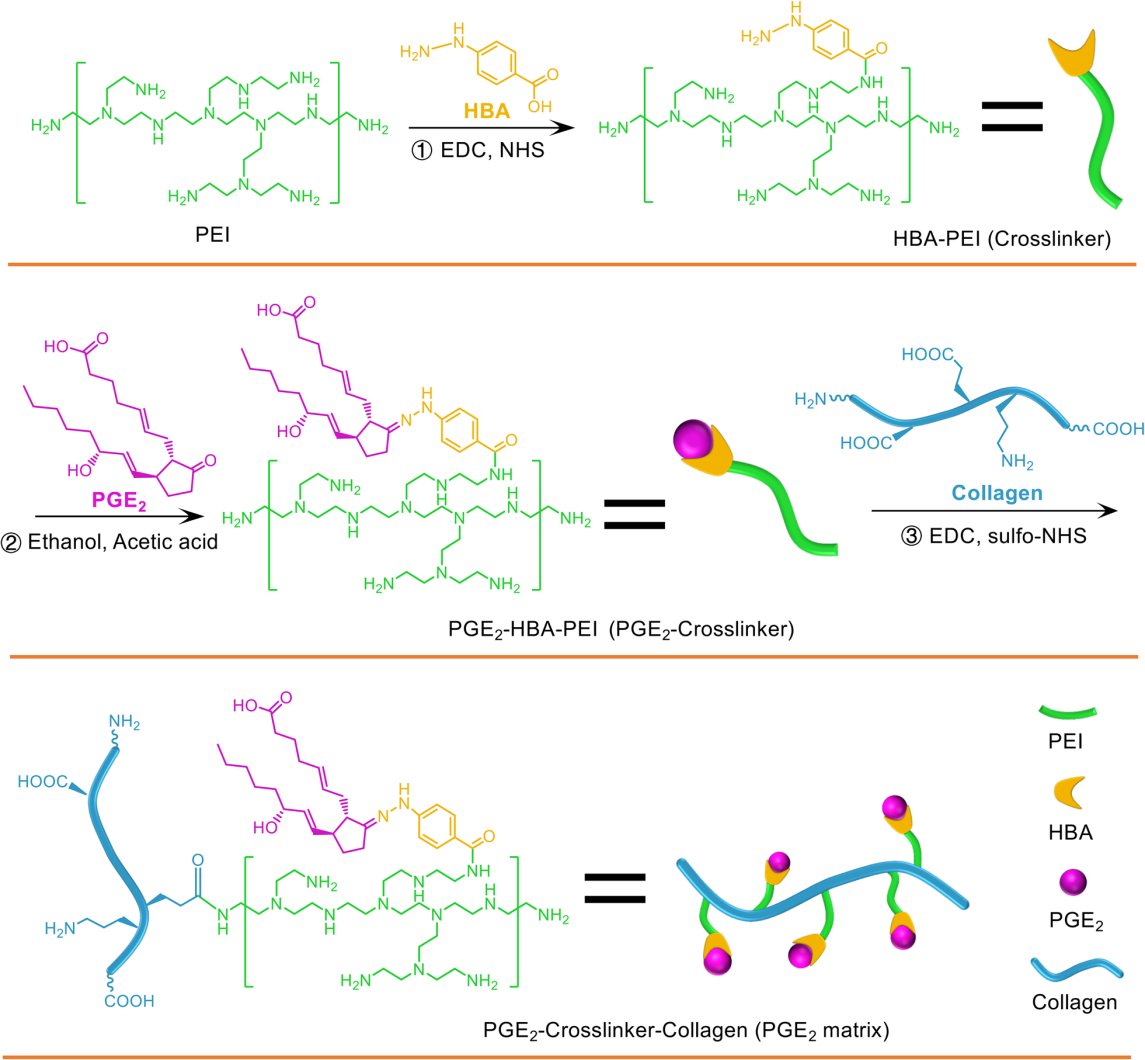
Schematic illustration of the design and preparation of the PGE_2_ matrix. Synthesis routes and schematic illustration of the crosslinker (HBA-PEI), the crosslinker-PGE_2_ and collagen-crosslinker-PGE_2_ (PGE_2_ matrix). *PEI* branched polyethylenimine, *EDC* 1-(3-dimethylaminopropyl)-3-ethylcarbodiimide hydrochloride, *NHS* *N*-hydroxy-succinimide

The PGE_2_ release collagen matrix (PGE_2_ matrix) was prepared using a three-step procedure, as illustrated in Fig. 1. The simplicity and versatility of hydrazone cross-linking have made it a strategy of choice for the conjugation of bioactive molecules [36]. Therefore, to build a slowly releasing system, we first synthesized the cross-linker (HBA-PEI), which was achieved by the reaction of an amino group on PEI with the carboxylic group on HBA through amidocarbonylation reactions. Then, PGE_2_ was further attached to the crosslinker via a pH-sensitive HBA linker to form hydrazone bonds. Therefore, as a final step, PGE_2_ could be cross-linked with a collagen scaffold by coupling the amine groups on the carboxyl groups of collagen to obtain the PGE_2_ matrix, which could slowly release PGE_2_ by gradually and slowly cleaving stable hydrazone bonds at neutral pH or slightly acidic condition.

**Fig. 2:**
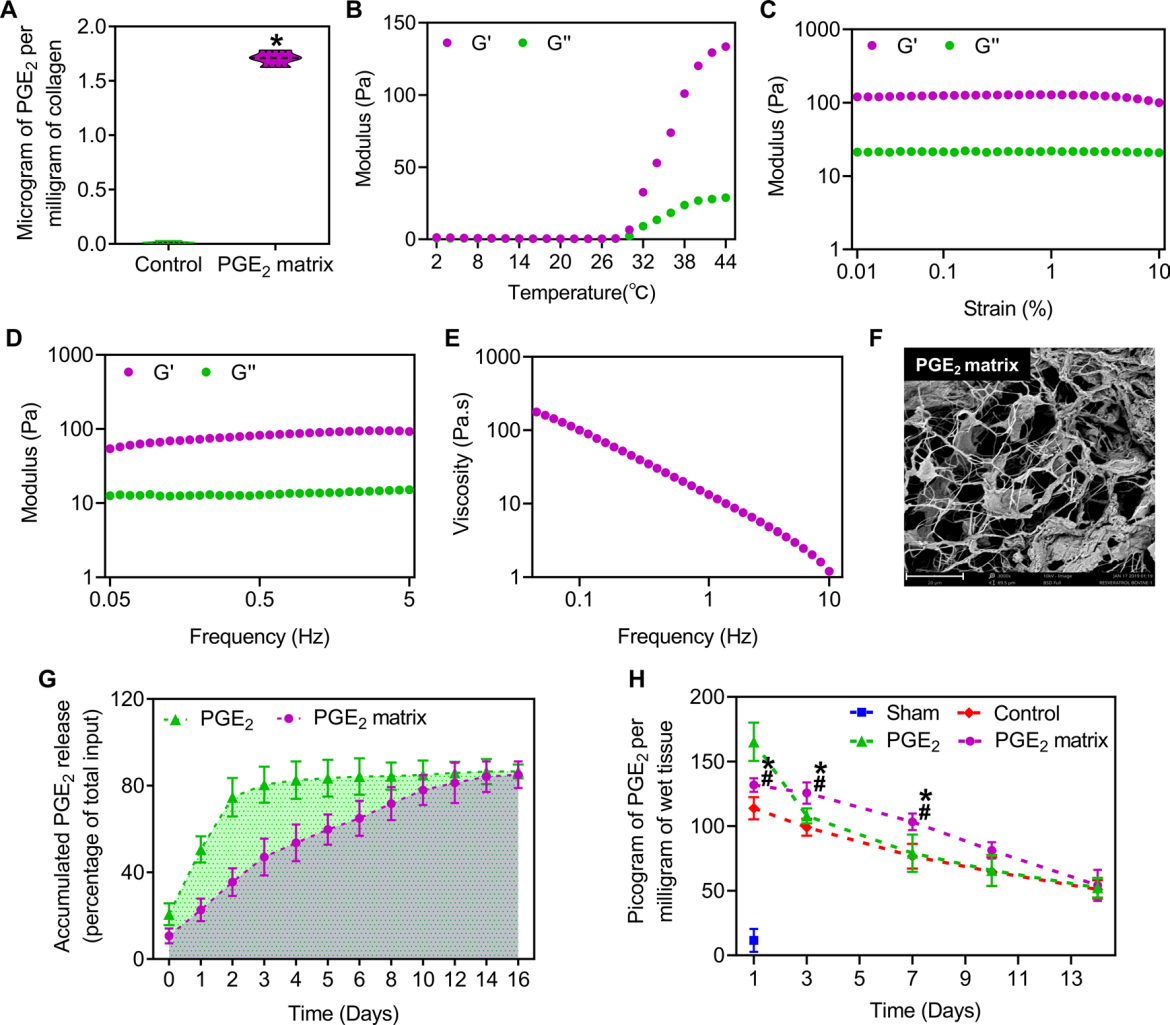
Characterization and PGE_2_ release kinetics of the PGE_2_ matrix. **A** Quantification of the level of PGE_2_ before and after crosslinking onto collagen using the ELISA assay. Pure collagen served as a control. Data are expressed as mean ± SD; *P < 0.05 versus control; The experiments were carried out in triplicate. Evaluation of the rheological profile of the PGE_2_ matrix with changes in temperature (**B**), strain sweep (**C**), and frequency sweep (**D**). G’ storage modulus and G’ loss modulus. **E** Complex viscosity vs. frequency plots for the PGE_2_ matrix. **F** A scanning electron micrograph (SEM) image of the lyophilized PGE_2_ matrix reveals the morphological structure. The bar represents 20 μm. **G** In vitro release profile of PGE_2_ from the PGE_2_ matrix determined by the ELISA assay. The PGE_2_ group (free PGE_2_ mixed with collagen) was tested as a control. Data are expressed as mean ± SD; the experiments were carried out in triplicate. **H** For in vivo PGE_2_ release analysis, the PGE_2_ matrix was injected into the hindlimb model of ischemic muscle. The muscle samples were then prepared and homogenized at different specific time points to assess the released PGE_2_ using an ELISA assay. Hindlimb ischemia mice injected with collagen served as a control group to measure signal change when injured. The Sham group served as a blank to reflect the baseline level of PGE_2_. Data are expressed as mean ± SD; n = 3, *P < 0.05 versus PGE_2_ group; ^#^*P* < 0.05 versus control

The results of the cell proliferation assay revealed that 2 μM was the optimal concentration of the PGE_2_ matrix (Additional file 1: Fig. S1A–C). The PGE_2_ content of the PGE_2_ matrix was 1.71 µg per milligram of collagen detected by the ELISA assay (Fig. 2A). The storage modulus (G′) and the loss modulus (G″) were monitored with an oscillatory time sweep at different temperatures. Based on the rheological measurements, our data revealed that the storage modulus (G′) of the PGE_2_ matrix clearly increased when the temperature increased to more than 30 °C (Fig. 2B), indicating the phase transition of the solution to gel. To test the mechanical properties of the PGE_2_ matrix, strain and frequency sweep tests were performed. The strain sweep showed that the PGE_2_ matrix poses higher G′ than G″ in the range of 0.01–10%, confirming its gel-like behavior (Fig. 2C). The frequency sweep test at a constant strain also showed that G′ presented higher values than G″ (Fig. 2D). Moreover, Fig. 2E showed that the complex viscosity of PGE_2_ matrix decreases linearly with increasing shear frequency. The morphological structure of the lyophilized PGE_2_ matrix was observed with the aid of scanning electron microscopy (SEM). Our results showed that the PGE_2_ matrix had interconnected pores with an average pore size of approximately 5 μm and was homogeneous (Fig. 2F).

### PGE_2_-releasing profile

To test whether the PGE_2_ matrix could release PGE_2_ for a prolonged time, we next examined the release kinetics of the PGE_2_ matrix and free PGE_2_ in collagen in vitro and in vivo using ELISA. The release kinetics of the accumulation of PGE_2_ in vitro are shown in Fig. 2G. We found that free PGE_2_ in collagen (PGE_2_ group) showed an apparent burst release at the first two to three days. In comparison, the PGE_2_ matrix group (crosslinked PGE_2_ on collagen) exhibited a release of more than 14 days. The release of PGE_2_ from different concentrations of the PGE_2_ matrix was further studied, and the results showed that different concentrations of the PGE_2_ matrix have very similar PGE_2_ release profiles (Additional file 1: Fig. S2A). Additionally, we next investigated the PGE_2_ release behavior of the PGE_2_ matrix under two different conditions, that is, pH 6.8 and 7.4. As shown in Additional file 1: Fig. S2B, the PGE_2_ matrix showed a relatively accelerated rate of PGE_2_ release in a more acidic environment (pH 6.8), while still showing sustained PGE_2_ release compared to uncrosslinked PGE_2_ (PGE_2_ group) under the same condition. Furthermore, the PGE_2_ release curve of the PGE_2_ matrix in vivo (Fig. 2H) showed that the level of PGE_2_ increased incrementally, which was consistent with the results in vitro. All of the above results indicated that the PGE_2_ matrix could gradually release PGE_2_ and extend its clearance time.

### Hemocompatibility and cytobiocompatibility of the PGE_2_ matrix in vitro

**Fig. 3:**
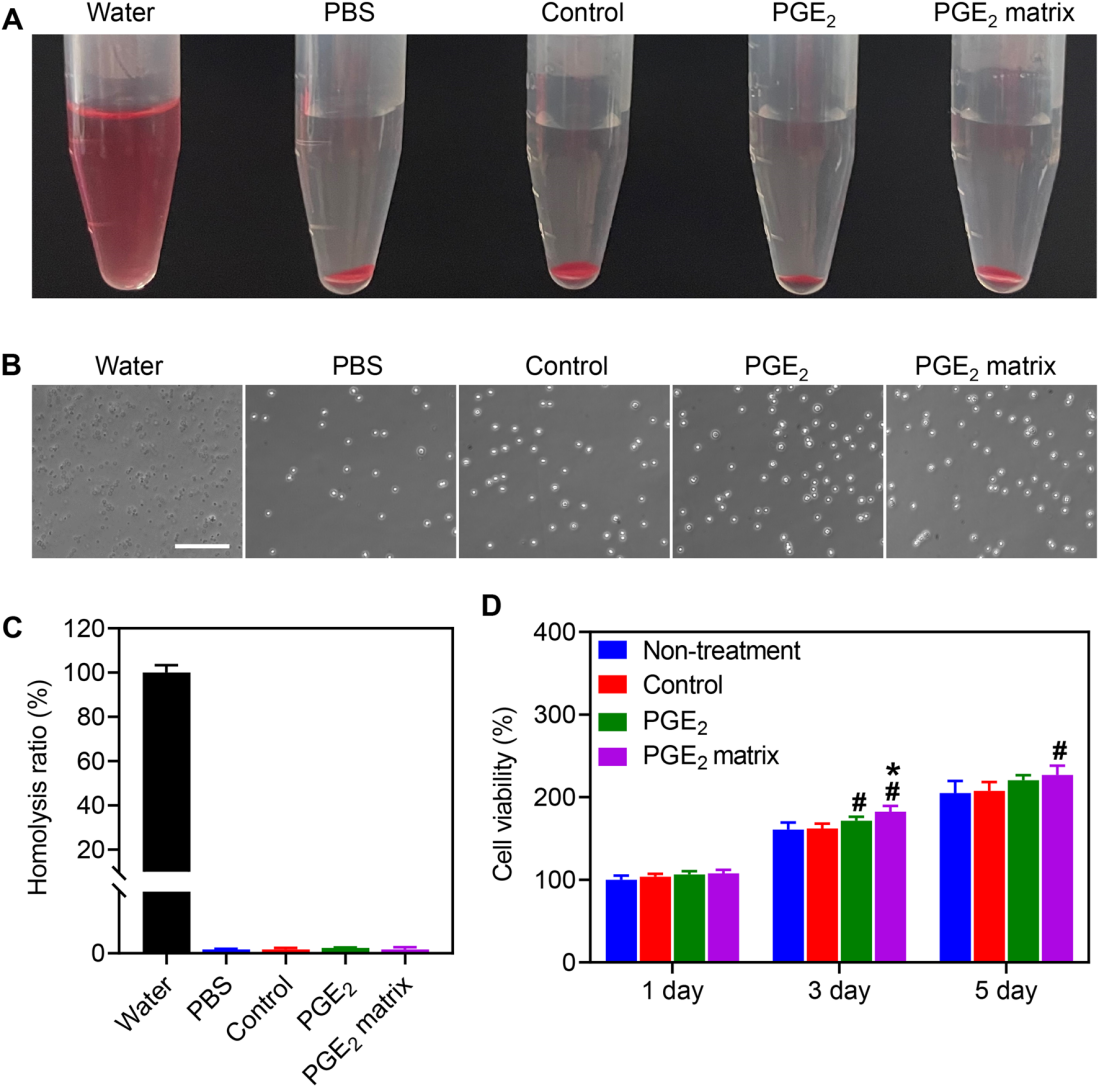
Hemocompatibility and cytocompatibility analysis of PGE_2_ matrix. **A** Overview of the red blood cell hemolysis assay; Red blood cells are incubated with Water (positive control), PBS (negative control), Control, PGE_2_, and PGE_2_ matrix. **B** Optical images of red blood cells. Scale bar, 200 μm. **C** Quantification of the hemolysis ratio. **D** Cell viability assay of HUVECs; which were treated with Control, PGE_2_, and PGE_2_ matrix for 1, 3, and 5 days. Data are expressed as mean ± SD; The experiments were carried out in triplicate. *P < 0.05 versus PGE_2_ group; ^#^*P* < 0.05 versus control

The hemocompatibility and cytotoxicity of the PGE_2_ matrix were assessed prior to use in experiments for HI treatment. The hemolytic test indicated that after incubation with Control, PGE_2_, and PGE_2_ matrix the cell integrality (Fig. 3A) and morphology (Fig. 3B) of red blood cells (RBCs) were similar to those of RBCs treated with negative control-PBS. As shown in Fig. 3C, the hemolytic ratio of all groups was less than 1%, which was much lower than the critical safe hemolysis rate for biomaterials (5%), indicating that the PGE_2_ matrix exhibited excellent blood compatibility. To investigate the cytotoxicity of the PGE_2_ matrix, HUVECs were seeded on various samples (Control, PGE_2_, and PGE_2_ matrix) for 1, 3, and 5 days, and the cell viability assay indicated that the PGE_2_ matrix also had good cytocompatibility (Fig. 3D). Overall, the good blood cell and endothelial cell compatibility endow the PGE_2_ matrix with great potential to improve the ischemic hindlimb and promote tissue regeneration.

### The PGE_2_ matrix possessed superior therapeutic efficacy for the treatment of HI

**Fig. 4:**
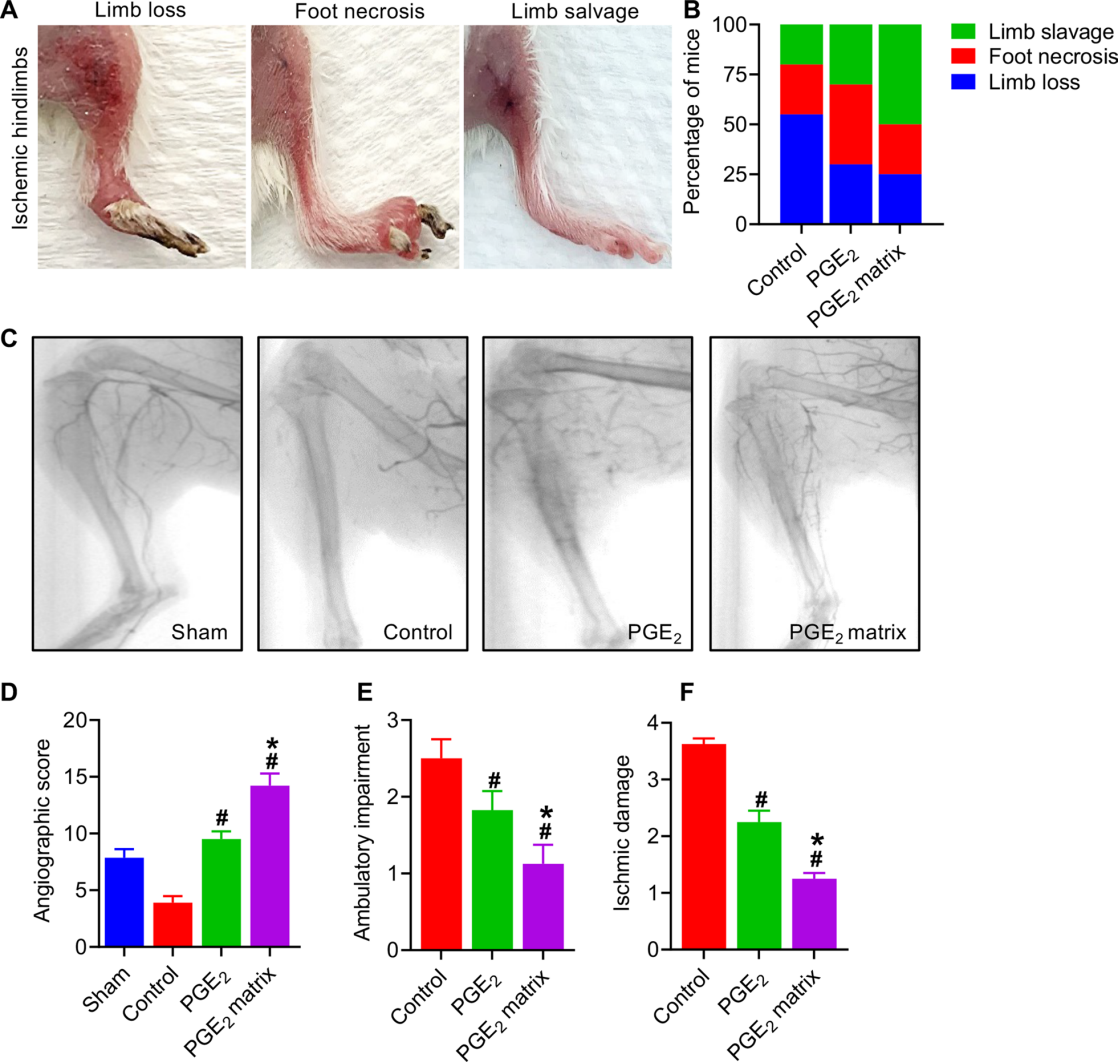
The PGE_2_ matrix possessed superior therapeutic efficacy for the treatment of HI. **A** Representative photographs of the ischemic limb, including salvage of the limb, foot necrosis, and loss of the limb on day 14 after ischemic treatment. **B** Quantification of the percentage of limb loss, foot necrosis, and limb salvage in treatment groups on day 14 (*n* = 8). **C** Representative photographs of angiography showing collateral vessel formation at ischemic sites on day 21. Mice were imaged in the prone position (*n* = 3). **D** Vascular density was determined by measuring the number of vessels in the unit area. Semiquantitative evaluation of ambulatory impairment (**E**) and ischemic damage (**F**) of hindlimb ischemia hindlimbs on day 14 in each group (*n* = 8). Data are expressed as mean ± SD. *P < 0.05 versus the PGE_2_ group; ^#^*P* < 0.05 versus the control group. Hindlimb ischemia mice injected with collagen as a control group

To evaluate the therapeutic efficacy of the PGE_2_ matrix, we monitored the outcome of the limb and collateral vessels development after ischemic injury. The results of the limbs were divided into three categories, including loss of limb, foot necrosis, and salvage of the limb (Fig. 4A). We comprehensively recorded and calculated different percentage distributions of outcomes among the three groups, as shown in Fig. 4B. Mice without effective treatment showed extensive necrosis of the ischemic hindlimb, resulting in an amputation rate of more than 55%, while treatment with PGE_2_ (32%) and the PGE_2_ matrix (23%) efficiently prevented limb loss. To investigate the development of collateral vessel in the limb after ischemia, we performed angiography in HI mice on day 21 after surgery. Angiographic images indicated that the PGE_2_ matrix significantly improved collateral vessel generation of collateral vessels at ischemia sites (Fig. 4C, D), suggesting that the PGE_2_ matrix has the potential to increase blood supply. Additionally, ambulatory impairment was semi-quantitatively measured to assess ischemic status. The PGE_2_ matrix relieved ambulatory and ischemic damage compared to other groups (Fig. 4E, F). All of these results indicated that the PGE_2_ matrix possessed superior therapeutic potential for the treatment of HI.

### The PGE_2_ matrix stimulated the angiogenesis of ischemic hindlimbs in vivo

**Fig. 5:**
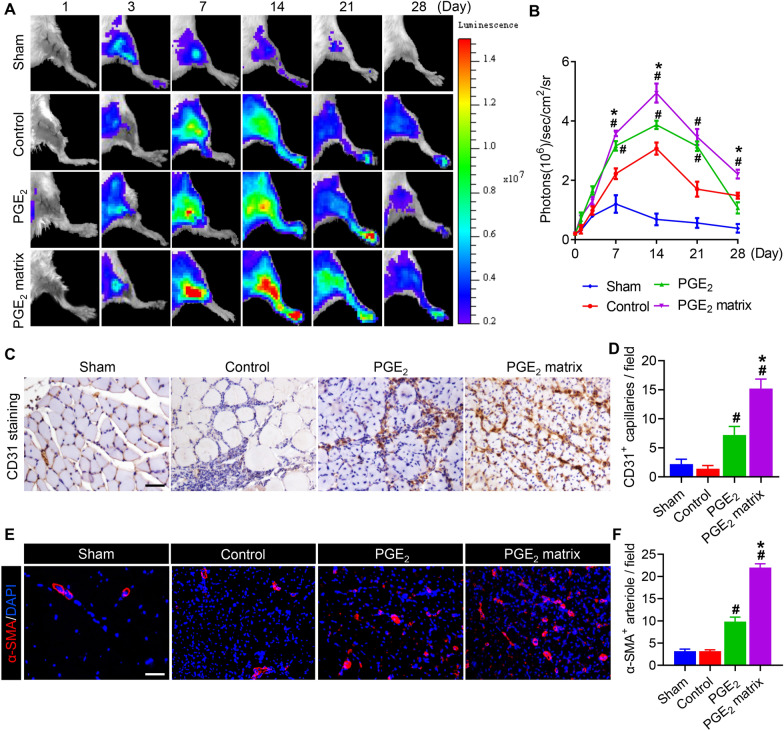
The PGE_2_ matrix stimulated angiogenesis of the ischemic hindlimbs in vivo. **A** Representative BLI images revealed the angiogenic tendency of ischemic tissues treated with the PGE_2_ matrix (*n* = 3). **B** Quantitative analysis of the Fluc signal in units of photons/s/cm^2^/steradian. **C** Immunohistochemical staining with CD31 on day 14 revealed capillaries in injured muscle sections (*n* = 5). The bar represents 100 μm. **D** Quantification of CD31 expression, angiogenesis-related marker in injured muscles. **E** Immunofluorescence staining with α-SMA on day 28 revealed arterioles at ischemic sites (*n* = 5). The bar represents 100 μm. **F** Quantification of immunostaining of angiogenesis-related makers α-SMA immunostaining in injured muscles. Data are expressed as mean ± SD. *P < 0.05 versus the PGE_2_ group; ^#^*P* < 0.05 versus the control group. Hindlimb ischemia mice injected with collagen as a control group

Angiogenesis, as a critical step in ischemic tissue regeneration after peripheral arterial disease, has a direct impact on the long-term effects associated with disease and mortality [37]. For real-time monitoring of angiogenesis at ischemia sites, VEGFR2-Fluc-KI transgenic mice were used to generate an HI mouse model and Fluc signals were captured using an IVIS living imaging system. The intensity of the signal represents angiogenesis directly because the expression of the Fluc gene is driven by the endogenous level of VEGFR2 [35]. The BLI signal was emitted and reached a peak on day 14 in all groups other than sham, and the strongest signal was detected in the PGE_2_ matrix group, indicating that the PGE_2_ matrix stimulated angiogenesis by activating the VEGF/VEGFR2 pathway (Fig. 5A, B). To further gain insight into the neovascularization of ischemic tissues after HI treatment with the PGE_2_ matrix, we performed an immunohistochemical examination after sacrificing mice on day 14 after surgery. Immunostaining for CD31 (Fig. 5C, D) revealed that the microvessel density increased significantly in the group treated with the PGE_2_ matrix. Moreover, α-SMA (Fig. 5E, F) immunofluorescent staining revealed that the PGE_2_ matrix significantly increased α-SMA^+^ mature blood vessels in the ischemic hindlimb. Histological results were consistent with BLI results, suggesting that the PGE_2_ matrix accelerated tissue regeneration through increased proangiogenic capacity.

### The PGE_2_ matrix improved the angiogenic effects in vitro

**Fig. 6:**
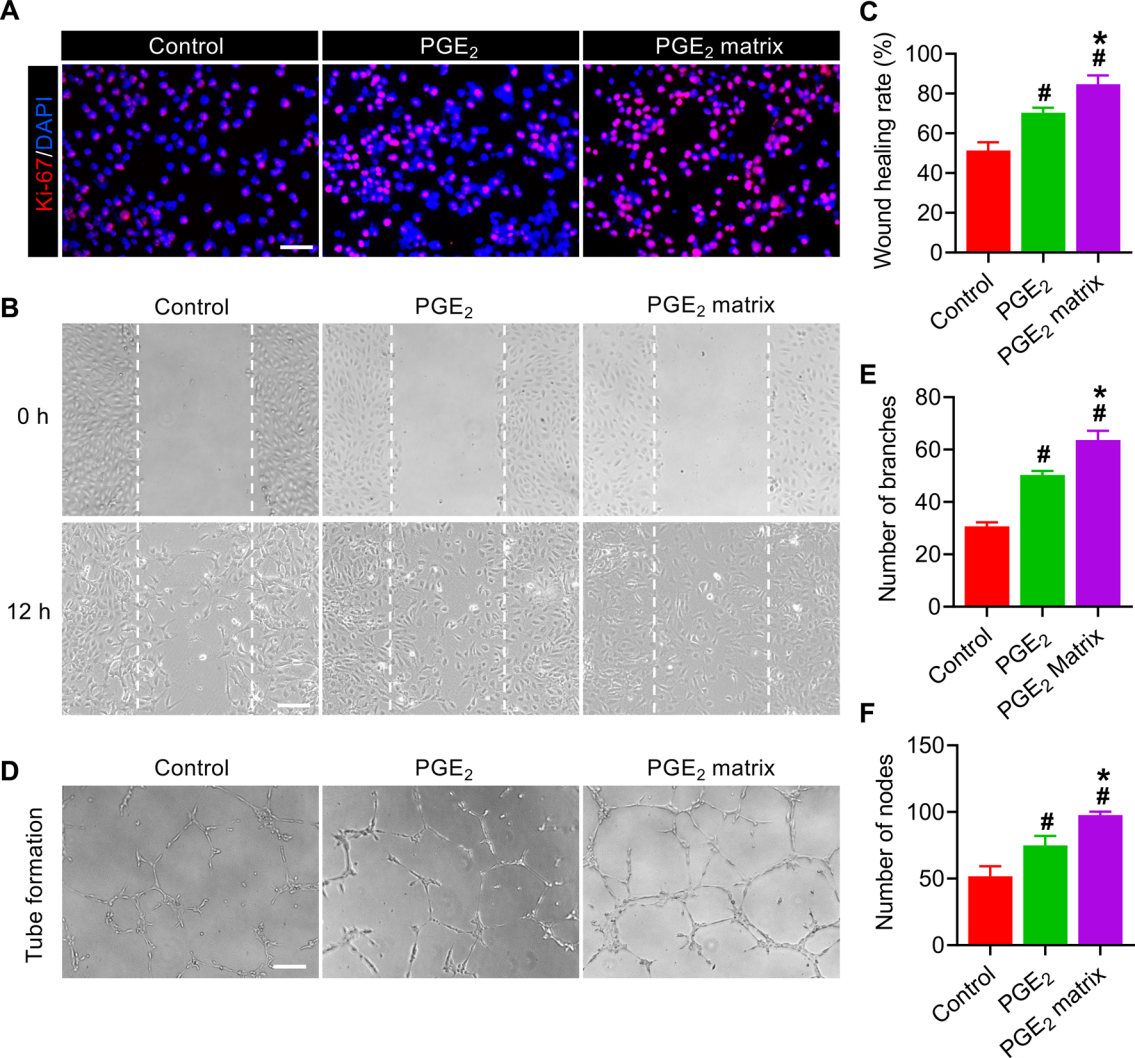
The PGE_2_ matrix improved the angiogenic effects of HUVECs in vitro. **A** Representative images of HUVECs treated with the PGE_2_ matrix for 48 h were stained with Ki-67. The scale bar represents 100 μm. **B** Images of the wound healing assay in HUVECs treated with PGE_2_ matrix for 0 and 12 h. The bar represents 200 μm. **C** The migration ratio of each group. **D** Images of the tube formation assay of HUVECs treated with the PGE_2_ matrix for 12 h. The bar represents 200 μm. Quantification of the branch number (**E**) and node number (**F**) in three random fields in each group. Data are expressed as mean ± SD. *P < 0.05 versus the PGE_2_ group; ^#^*P* < 0.05 versus the control group. HUVECs cultured on a collagen-coated plate as a control group. The experiments were carried out in triplicate

To examine the proangiogenic potential of the PGE_2_ matrix on endothelial cells in vitro, we performed a proliferation assay, a tube formation assay and a scratch wound healing assay. The promoting effect of the PGE_2_ matrix on HUVECs was evaluated by immunofluorescence staining of Ki-67-proliferating markers. The results revealed that the PGE_2_ matrix significantly stimulated the proliferation of HUVECs compared to other groups (Fig. 6A). Next, we investigate whether the PGE_2_ matrix affects the proangiogenic effects of HUVECs. Cell migration was examined by a scratch wound healing assay. After being treated with the PGE_2_ matrix for 12 h, the wound closure ratio (84.6%) was markedly higher than that treated with the control group (51.3%) and the PGE_2_ group (70.3%) (Fig. 6B, C). Regarding the tube formation assay, the number of branches and nodes, HUVEC tubes increased significantly with pretreatment of the PGE_2_ matrix, suggesting a stronger proangiogenic capacity (Fig. 6D–F). All results implied that the PGE_2_ matrix exerts promotive effects on the angiogenic capacities of HUVECs.

### The protective effects of the PGE_2_ matrix in vitro

**Fig. 7:**
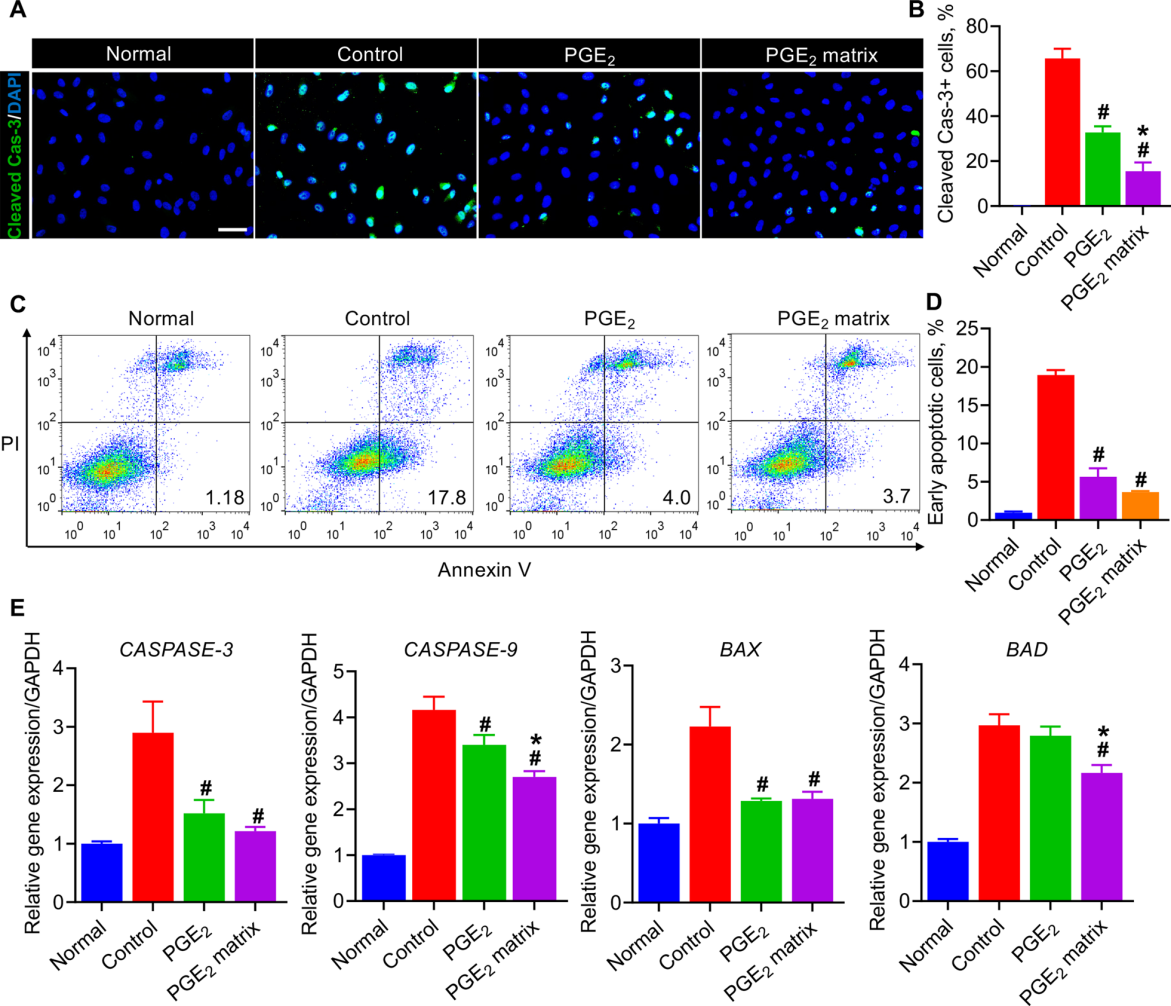
The PGE_2_ matrix promoted angiogenesis by decreasing apoptosis of HUVECs in vitro. **A** Representative images showed apoptosis (cleaved caspase-3, green) of HUVECs. Scale bar, 100 μm. **B** Quantification of immunostaining of cleaved caspase-3. **C** Flow cytometry of annexin V and PI. **D** Quantification of apoptotic cells. **E** Real-time PCR analysis of HUVEC gene expression related to apoptosis. The HUVECs were cultured in plates coated with PGE_2_ matrix for 48 h and then treated with 200 μM H_2_O_2_ for 6 h. Data are expressed as mean ± SD. *P < 0.05 versus the PGE_2_ group; ^#^*P* < 0.05 versus the control group. HUVECs cultured on a collagen-coated plate treated with H_2_O_2_ as a control group. The experiments were carried out in triplicate

Postischemia, mitochondrial injury in skeletal muscle cells sparks the generation of reactive oxygen species (ROS), leading to vascular endothelial cell apoptosis and further damage to angiogenesis in the muscle regeneration process [4, 38]. Therefore, next we mimicked the challenge of oxidative stress in vitro using H_2_O_2_ to assess the antiapoptotic effects of the PGE_2_ matrix on HUVECs. The protein level of cleaved caspase-3, a key apoptosis molecule, was detected by immunofluorescence staining (Fig. 7A, B). Annexin V-FITC/PI double labeling combined with flow cytometry was performed to detect the early rate of apoptosis (Fig. 7C, D). Furthermore, apoptosis-related gene expressions, including *CASPASE-3*, *CASPASE-9*, *BAX*, and *BAD*, were confirmed by quantitative real-time RT-PCR (Fig. 7E). It can be seen from the results that the control group significantly activated the expression of the cleaved caspase-3 protein and increased the level of genes related to early apoptosis. In conclusion, the PGE_2_ matrix could significantly improve the apoptosis of HUVECs induced by H_2_O_2_. These results demonstrated that the PGE_2_ matrix protects against endothelial cell damage by diminishing apoptosis in vitro.

### The PGE_2_ matrix exhibits more predominance in promoting MyoD1-mediated skeletal muscle regeneration

**Fig. 8:**
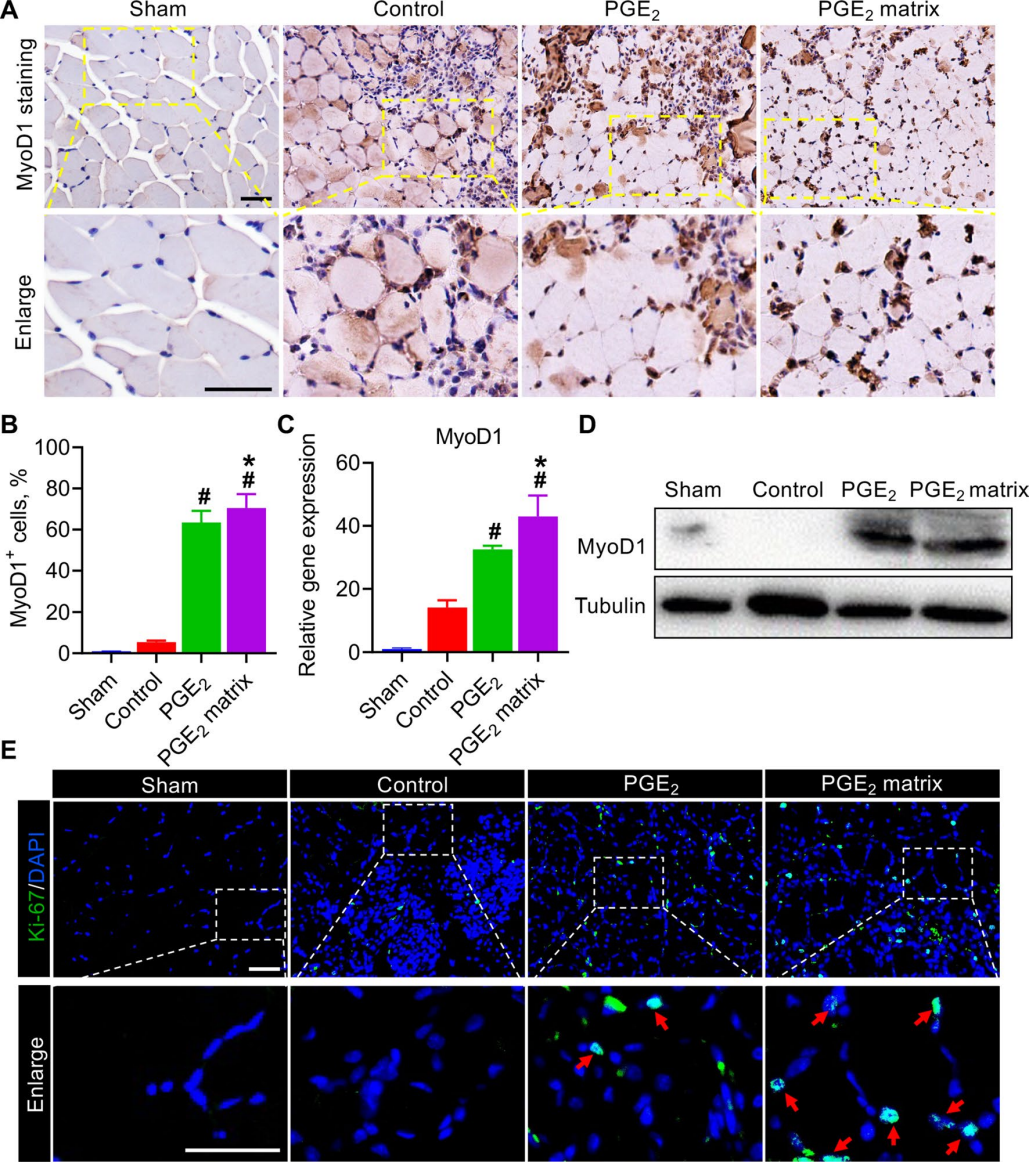
The PGE_2_ matrix improved MyoD1-mediated skeletal muscle regeneration. **A** Representative images of immunohistochemical staining of MyoD1 in ischemic limbs with different treatment on day 7. **B** Quantification of MyoD1 immunostaining in injured muscles. **C** Expression of the MyoD1 gene in ischemic muscle with different treatment on day 7. **D** Western blot analysis of MyoD1 expression of MyoD1 in injured muscles on day 7. **E** Representative images of ischemic limbs with different treatment on day 7 stained for Ki-67. The red arrowheads highlighted Ki-67 + muscle cells. Data are expressed as mean ± SD. *n* = 5. Scale bar, 100 μm. *P < 0.05 versus the PGE_2_ group; ^#^*P* < 0.05 versus the control group. Hindlimb ischemia mice injected with collagen as a control group

We confirm that the PGE_2_ matrix exerts a more superior proangiogenic function compared to other groups; In addition to recovery of blood supply, regeneration of skeletal muscle regeneration [8] is a crucial therapeutic target that needs to be considered. Myogenic regulatory factor, MyoD1, is a marker of the differentiation and proliferation of satellite cells of skeletal muscle, which make a valuable contribution to muscle regeneration [39]. Therefore, to clarify the underlying mechanism, the expression of MyoD1 at injured sites at different time points after surgery was investigated in subsequent investigations. Immunohistochemical analysis revealed that the PGE_2_ matrix could significantly increase MyoD1 expression over other groups on day 7 after surgery, reflecting enhanced skeletal muscle regeneration during the early stages (Fig. 8A, B). MyoD1 expression was further evaluated in representative samples using Western blot analysis and real-time PCR analyzes (Fig. 8C). The results showed a pattern of expression of MyoD1 consistent with that observed by the immunohistochemical assay (Fig. 8D). However, on day 14, a decrease in MyoD1 level was observed in regenerated muscles treated with the PGE_2_ matrix (Additional file 1: Fig. S3A–C), which coincides with previous findings [40], implying that the PGE_2_ matrix accelerated the time course of MyoD1 and further promoted the completion of muscle regeneration. Furthermore, the proliferation-associated factor, Ki-67, at the protein level, as evidenced by immunofluorescence analyzes, points to increased cellular proliferation in the response of the PGE_2_ matrix to ischemic limbs (Fig. 8E). The above results indicated that sustained release of PGE_2_ exhibits more predominance in promoting MyoD1-mediated muscle regeneration.

### The PGE_2_ matrix promoted the recovery of hindlimb function

**Fig. 9:**
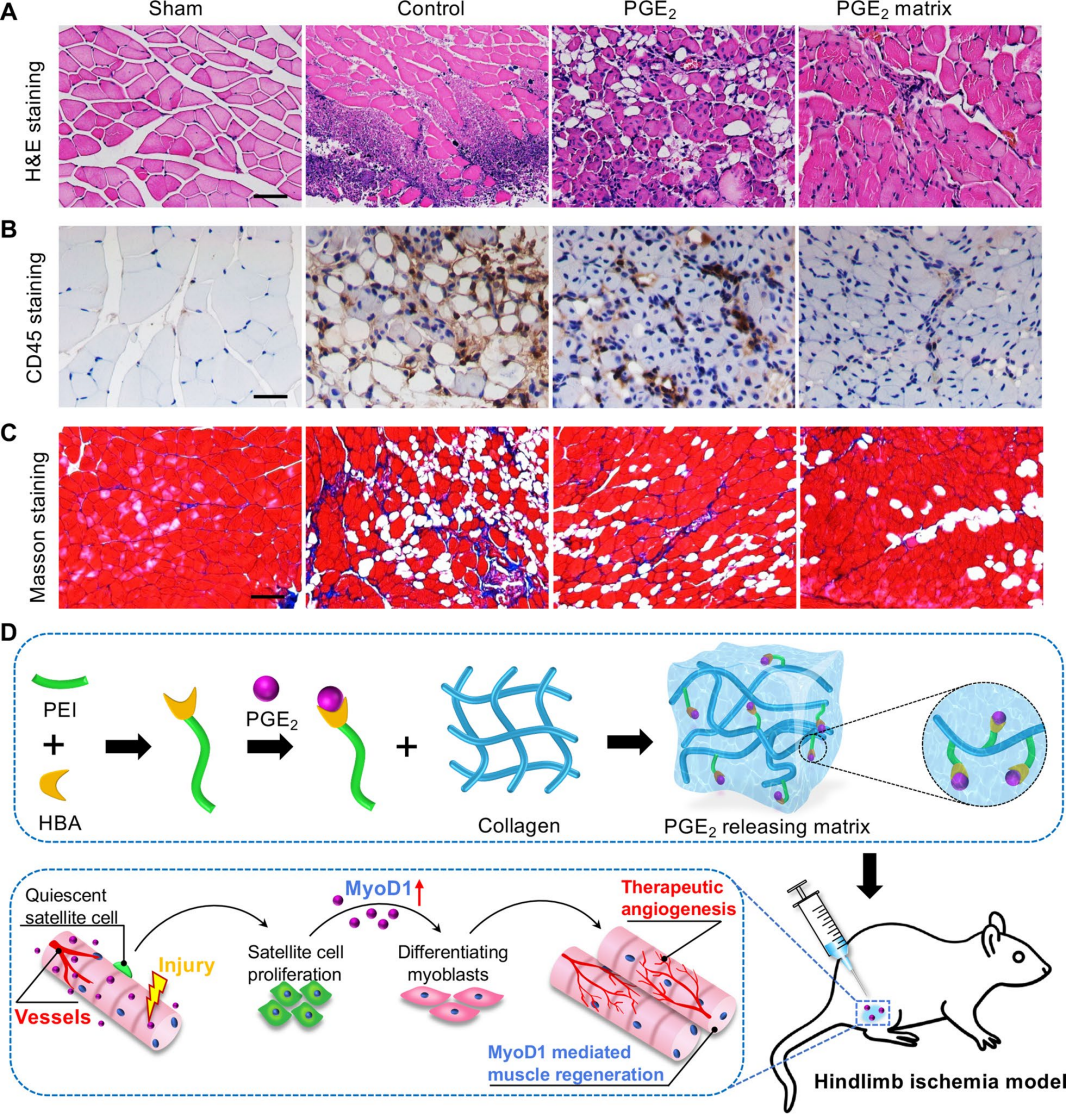
The PGE_2_ matrix promoted recovery of hindlimb function. **A** Representative H&E-stained images of muscle tissue with different treatment on day 14. *n* = 5. Scale bar, 100 μm. **B** Representative images of anti-CD45 immunohistochemical staining in injured muscles on day 14. *n* = 5. Scale bar, 100 μm. **C** Masson’s trichrome staining of muscle sections on day 28. *n* = 5. Scale bar, 100 μm. **D** Schematic diagram of the PGE_2_ release matrix for muscle regeneration. Preparation of the PGE_2_ matrix to extend the release of PGE_2_ with therapeutic potential to treat hindlimb ischemia by promoting neovascularization and MyoD1-mediated regeneration of skeletal muscle

To further investigate the therapeutic potential of the PGE_2_ matrix, we proceeded to evaluate the morphological changes in muscle tissues using histological examination. We first euthanized mice from each group to collect hindlimb tissues on day 14 and perform hematoxylin and eosin (H&E) staining to examine the severity of the injury. H&E staining of ischemic limb muscles illustrated that the PGE_2_ matrix significantly alleviated muscle fiber degeneration and necrosis, inflammatory cell infiltration, and fat deposition in injured tissue (Fig. 9A) and markedly increased the area of normal muscle fibers (Additional file 1: Fig. S4A). Infiltration of inflammatory cells has been shown to be a critical factor leading to muscle cell apoptosis and further limits recovery in ischemic muscle injury [41]. Infiltration of inflammatory cells was evaluated by immunostaining with CD45-leukocyte markers and F4/80-macrophage markers. Representative photographs are shown in Fig. 9B and Additional file 1: Fig. S5A. The staining results revealed that the PGE_2_ matrix treatment had minimal inflammatory cell infiltration (Additional file 1: Fig. S4B, S5B) and further alleviated muscle cell apoptosis (Additional file 1: Fig. S5C, D). The fibrotic areas identified by Masson’s trichrome staining on day 28 also confirmed that the PGE_2_ matrix decreased fibrosis (Fig. 9C; Additional file 1: Fig. S4C). All of these data indicate that a more pronounced effect and an extensive muscle protection effect were observed in the PGE_2_ matrix group.

## Discussion

This study demonstrates that the PGE_2_ release matrix effectively improves functional recovery from hindlimb ischemia. In the present study, we characterize a new injectable collagen matrix generated by chemically bonding PGE_2_ to the collagen matrix as a delivery system to extend the release of PGE_2_. We found that the PGE_2_ matrix markedly increased the half-life of PGE_2_ in vitro and in vivo, which is an attractive therapeutic strategy for effective and safe regeneration. Subsequently, the therapeutic effects of the PGE_2_ matrix in treating hindlimb ischemia were evaluated. Our findings revealed that the administration of the PGE_2_ matrix improved tissue blood supply by stimulating angiogenesis and improving microcirculation, while further promoting skeletal muscle regeneration by stimulating MyoD1-mediated skeletal myogenesis (Fig. 9D).

The bioactive molecule PGE_2_ has been proposed to be a critical mediator in the regulation of angiogenesis, immune regulation, and tissue regeneration [13, 15, 42, 43]. However, the clinical application of PGE_2_ is hampered by its short half-life in circulation and the lack of viable systems that demonstrate sustained release of PGE_2_. Synthetic or natural materials, including collagen, gelatin, alginate, chitosan, and hyaluronic acid, have emerged as a popular material for the delivery of bioactive molecules, while limited to physical incorporation with bioactive molecules [34, 44, 45]. For example, incorporating PGE_2_ with chitosan hydrogel has been shown to extend the half-life of PGE_2_ and further contribute to wound healing [15]. Furthermore, nanosized hydrogel encapsulation also allowed PGE_2_ to have a prolonged half-life and exert positive effects on bone therapy [46]. However, physical incorporation of PGE_2_ with hydrogels does provide a relatively effective strategy for prolonged release of PGE_2_, whereas it may result in a sudden, fast and uncontrolled release immediately after administration [47]. Our study describes a delivery system to control the sustained release of PGE_2_ from collagen scaffolds by chemical cross-linking to further delay the release rate of PGE_2_, which is innovative and different from other similar studies. The simplicity and versatility of hydrazone cross-linking have made it a strategy of choice for the conjugation of bioactive molecules [36]. Hence, we first synthesized the cross-linker (HBA-PEI) achieved by the reaction of an amino group on PEI with the carboxylic group on HBA through amidocarbonylation reactions. Then, PGE_2_ was further attached to the cross-linker via a pH-sensitive HBA linker, and the hydrazone bonds would exhibit a decelerated release at neutral pH or a slightly acidic pathological microenvironment. Finally, the cross-linker carrying PGE_2,_ which was grafted onto collagen by a dehydration condensation reaction to obtain the PGE_2_ matrix, which could slowly release PGE_2_ slowly. Furthermore, we have shown that the PGE_2_ matrix exerted more superior extending the clearance time of PGE_2_ in vivo and in vitro. The presented PGE_2_ matrix has great potential for use as bioactive small molecule delivery carriers for the stimulation of therapeutic neovascularization and functional regeneration of ischemic tissues.

The main pathophysiology of critical limb ischemia (CLI) is vascular blockage of the peripheral limbs resulting from arteriosclerosis and subsequent damage to skeletal muscle, which ultimately leads to loss of function of the limbs [48]. Consequently, to treat this ischemic disease, it is crucial to regenerate the damaged skeletal muscle simultaneously with angiogenesis. It is well established that PGE_2_ is a promising bioactive small molecule to induce angiogenesis and improve skeletal muscle regeneration after ischemic injury. However, the potential biphasic effects of PGE_2_ have been reported in tissues at low and high levels [49]. Lower concentrations (nM) of PGE_2_ can enhance cell proliferation, promote angiogenesis, and maintain cell differentiation homeostasis, while higher concentrations (μM) decrease cell proliferation, induce inflammation and apoptosis, and can be detrimental to tissue regeneration [50]. Therefore, the slow-release PGE_2_ delivery system in the present study has the potential to avoid counterproductive outcomes from burst release and deliver the appropriate dose to the injured site. Furthermore, our findings highlighted that PGE_2_ matrix therapeutics had superior beneficial effects on structural and functional recovery in a mouse model of hindlimb ischemia.

The non-invasive exploration of various biological processes achieved by reporter gene imaging strategies is a growing molecular imaging modality to longitudinally study target factor expression, which could offer direct evidence for the elucidation of therapeutic mechanisms [51]. This strategy that combined a reporter probe with a specific imaging device detected the accumulation of specific signals, which are induced by a reporter protein in living subjects [52]. Angiogenesis, the formation of new capillaries, which is essential for most physiological processes, including tissue regeneration after ischemic injury [53, 54]. A cascade of cellular events was involved in this complicate biological process, mainly including degradation of the extracellular matrix, proliferation, migration, and tube formation of vascular endothelial cells, and vessel maturation [55–57]. Vascular endothelial growth factor (VEGF) and its receptor VEGFR2 are considered potent and important targets that regulate angiogenesis [54, 58]. In this study, real-time evaluation of angiogenesis processes involved in tissue regenerative mechanisms by visualization of VEGFR2 expression in vivo. Based on the findings of BLI, an obvious promotion in angiogenesis was observed in the PGE_2_ group and the PGE_2_ matrix group, suggesting that PGE_2_ could strikingly up-regulate the VEGF/VEGFR2 pathway, further resulting in functional recovery after ischemia. Furthermore, the PGE_2_ matrix could provide sustained release of PGE_2_, resulting in superior proangiogenic effects on hindlimb ischemia. Furthermore, our results revealed that the PGE_2_ matrix promoted cell proliferation, migration, and vessel-like formation in HUVECs, which underlies its critical role in angiogenesis.

In addition to improving the ischemic environment, skeletal muscle regeneration is a beneficial and crucial therapeutic target that should be considered. Skeletal muscle regeneration is considered to recapitulate myogenesis in some respects [59]. In response to ischemic injury, skeletal muscle-specific stem cells, called satellite cells (SCs), enter the cell cycle and initiate the repair process that basically recapitulates myogenesis, a process regulated by MyoD1 [60]. MyoD1, as a myogenic regulatory factor, could induce SC proliferation and differentiation, which make a valuable contribution to myogenesis and further improve muscle repair [39]. MyoD1 expression was induced on day 1 after injury, denoting the beginning of muscle regeneration, and peaked on day 7 [61]. Furthermore, previous studies demonstrated that PGE_2_ could accelerate myogenic differentiation of skeletal muscle by upregulating MyoD1 levels [21, 62, 63]. Our findings showed that the PGE_2_ matrix accelerated the time course of MyoD1-mediated myogenesis along with skeletal muscle regeneration in vivo by increasing MyoD1 levels on day 7 after injury. The improved muscle regeneration response observed in this study may result from extending the half-life of PGE_2_ by crosslinking PGE_2_ to the collagen matrix, which could explain the great therapeutic function of the PGE_2_ matrix in the treatment of hindlimb ischemia.

Engineered matrix with bioactive molecule could prolong the release of regenerative factors and improve its local retention for excellent tissue regeneration [64, 65]. Therefore, this slow-release delivery strategy for the sustained effects of PGE_2_ provides a practical idea for clinical application. In our study, we investigated the superior therapeutic efficacy of the PGE_2_ matrix in an ischemic hindlimb model, which may facilitate the development of PGE_2_-based therapies to significantly improve tissue regeneration. Moreover, we utilized collagen as a scaffold to form the PGE_2_ matrix that has good biosafety and relatively low economic cost. More importantly, per single injection in vivo can achieve an ideal therapeutic effect without side effects, indicating that the PGE_2_ matrix is a feasible, effective, and durable therapy with potential clinical application.

## Conclusion

Overall, the work presented here demonstrated a new injectable collagen matrix generated by chemically bonding PGE_2_ to collagen scaffolds as a delivery system to extend the release of PGE_2_ with enhanced therapeutic effects. This PGE_2_ matrix could improve tissue blood supply through increased functional angiogenesis, inhibit inflammatory cell recruitment, and promote MyoD1-mediated regeneration of skeletal muscle, resulting in better muscle function. The novel strategy can serve as a generic delivery system to improve the clinical application of bioactive small molecules for regeneration medicine.

## Supplementary Information

**Additional file 1: Table S1**. The sequences of human primers. **Table S2**. The sequences of mouse primers. **Fig. S1. **The PGE_2_ matrix promoted HUVECs proliferation in optimal concentration. **Fig. S2. **PGE_2_ release kinetics from the PGE_2_ matrix. **Fig. S3. **The time course of MyoD1 in ischemic muscle tissues treated with the PGE_2_ matrix. **Fig. S4. **PGE_2_ matrix ameliorated tissue injury in ischemic hindlimbs. **Fig. S5. **PGE_2_ matrix inhibited inflammatory responses and prevented apoptosis in vivo. **Fig. S6. **Images of the uncropped immunoblots shown in Fig. 7D. Boxes indicate cropped regions.

## Data Availability

Not applicable.

## Abbreviations

CLI: Critical limb ischemia
COX: Cyclooxygenase
HI: Hindlimb ischemia
HUVECs: Human umbilical vein endothelial cells
Myf5: Myogenic factor 5
MyoD1: Myoblast determination protein
PAD: Peripheral artery disease
PGE_2_: Prostaglandin E_2_
SCs: Satellite cells
