# Supplementary material for: The sustained PGE2 release matrix improves neovascularization and skeletal muscle regeneration in a hindlimb ischemia model
Source: J Nanobiotechnology. 2022 Feb 24;20:95. doi: 10.1186/s12951-022-01301-3 (PMC8867652; doi:10.1186/s12951-022-01301-3)
Supplement: Supplementary file 1 — Additional file 1: Table S1. The sequences of human primers. Table S2. The sequences of mouse primers. Fig. S1. The PGE2 matrix promoted HUVECs proliferation in optimal concentration. Fig. S2. PGE2 release kinetics from the PGE2 matrix. Fig. S3. The time course of MyoD1 in ischemic muscle tissues treated with the PGE2 matrix. Fig. S4. PGE2 matrix ameliorated tissue injury in ischemic hindlimbs. Fig. S5. PGE2 matrix inhibited inflammatory responses and prevented apoptosis in vivo. Fig. S6. Images of the uncropped immunoblots shown in Fig. 7D. Boxes indicate cropped regions. [file 12951_2022_1301_MOESM1_ESM.docx]

**The Sustained PGE_2_ Release Matrix Improves Neovascularization and Skeletal Muscle Regeneration in a Hindlimb Ischemia Model**

Haoyan Huang, Shang Chen, Hui Cheng, Jiasong Cao, Jun Zhang, Yuqiao Chang, Xiaohong Shen, Zhikun Guo, Zhibo Han, Guoqiang Hua, Zhong-Chao Han, Nadia Benkirane-Jessel, Ying Chang, Zongjin Li

**Supplemental Table 1**. The sequences of human primers.

| Transcription | Primer sequences |
| --- | --- |
| CASPASE-3 | Forward: TGCATACTCCACAGCACCTG  Reverse: TCTGTTGCCACCTTTCGGTT |
| CASPASE-9 | Forward: GCAAGCAGCAAAGTTGTCGA  Reverse: GGACTCACGGCAGAAGTTCA |
| BAX | Forward: AAGGTGCCGGAACTGATCAG  Reverse: AAAGTAGGAGAGGAGGCCGT |
| BAD | Forward: AGAGTTTGAGCCGAGTGAGC  Reverse: ATGATGGCTGCTGCTGGTT |
| GAPDH | Forward: GGAGCGAGATCCCTCCAAAAT  Reverse: GGCTGTTGTCATACTTCTCATGG |

**Supplemental Table 2**. The sequences of mouse primers.

| Transcription | Primer sequences |
| --- | --- |
| MyoD1 | Forward: CCACTCCGGGACATAGACTTG  Reverse: AAAAGCGCAGGTCTGGTGAG |

**Supplementary Figures & Legends**


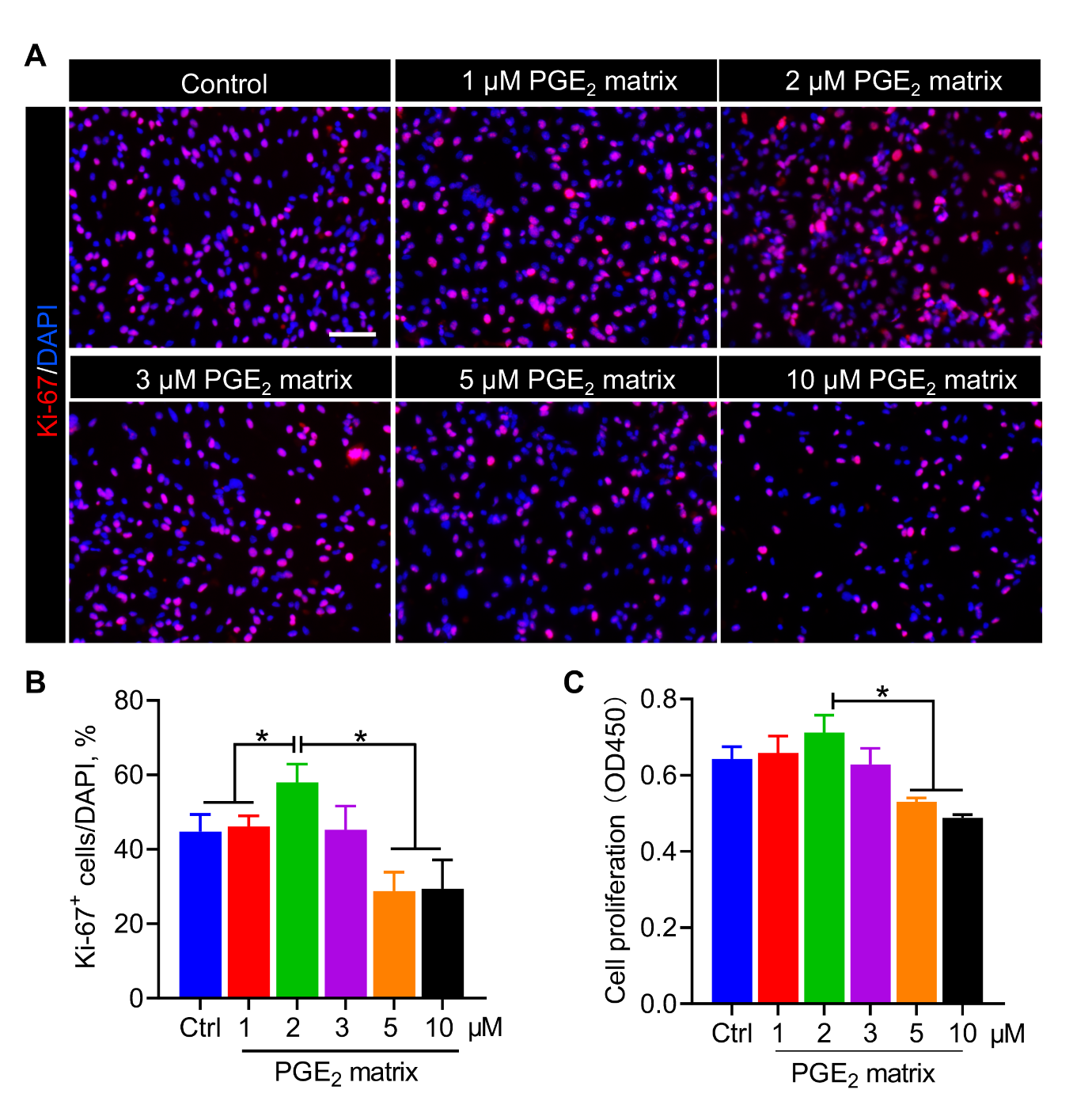


**Supplementary Fig. 1 The PGE_2_ matrix promoted HUVECs proliferation in optimal concentration. (A)** Representative images showed the proliferation (Ki-67, red) of HUVECs treated with different concentrations of the PGE_2_ matrix. **(B)** Quantification of Ki-67 immunostaining of HUVECs. **(C)** The CCK-8 assay showed the proliferation of HUVECs with different concentrations of PGE_2_ matrix. Data are expressed as the mean ± SD. Scale bar 100 μm. The experiments were performed in triplicate.

| **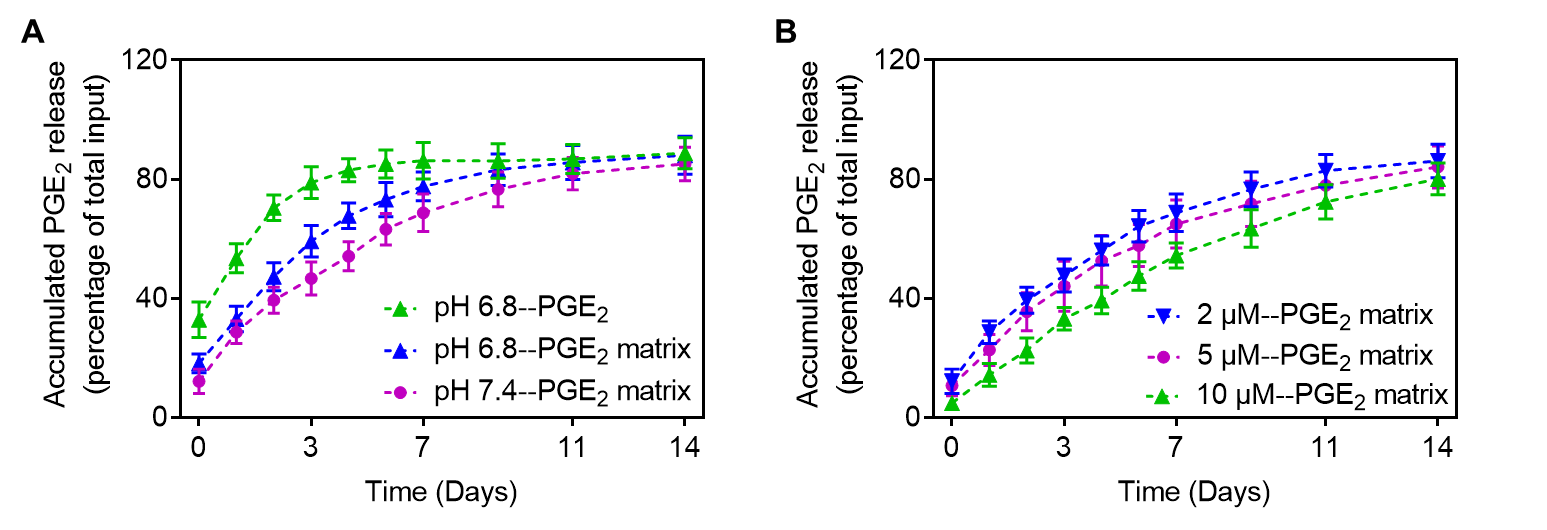** |
| --- |
| **Supplementary Fig. 2 PGE_2_ release kinetics from the PGE_2_ matrix. (A)** The PGE_2_ release profiles from PGE_2_ matrix with different concentrations. **(B)** The PGE_2_ release profiles from PGE_2_ matrix under different pH conditions. |


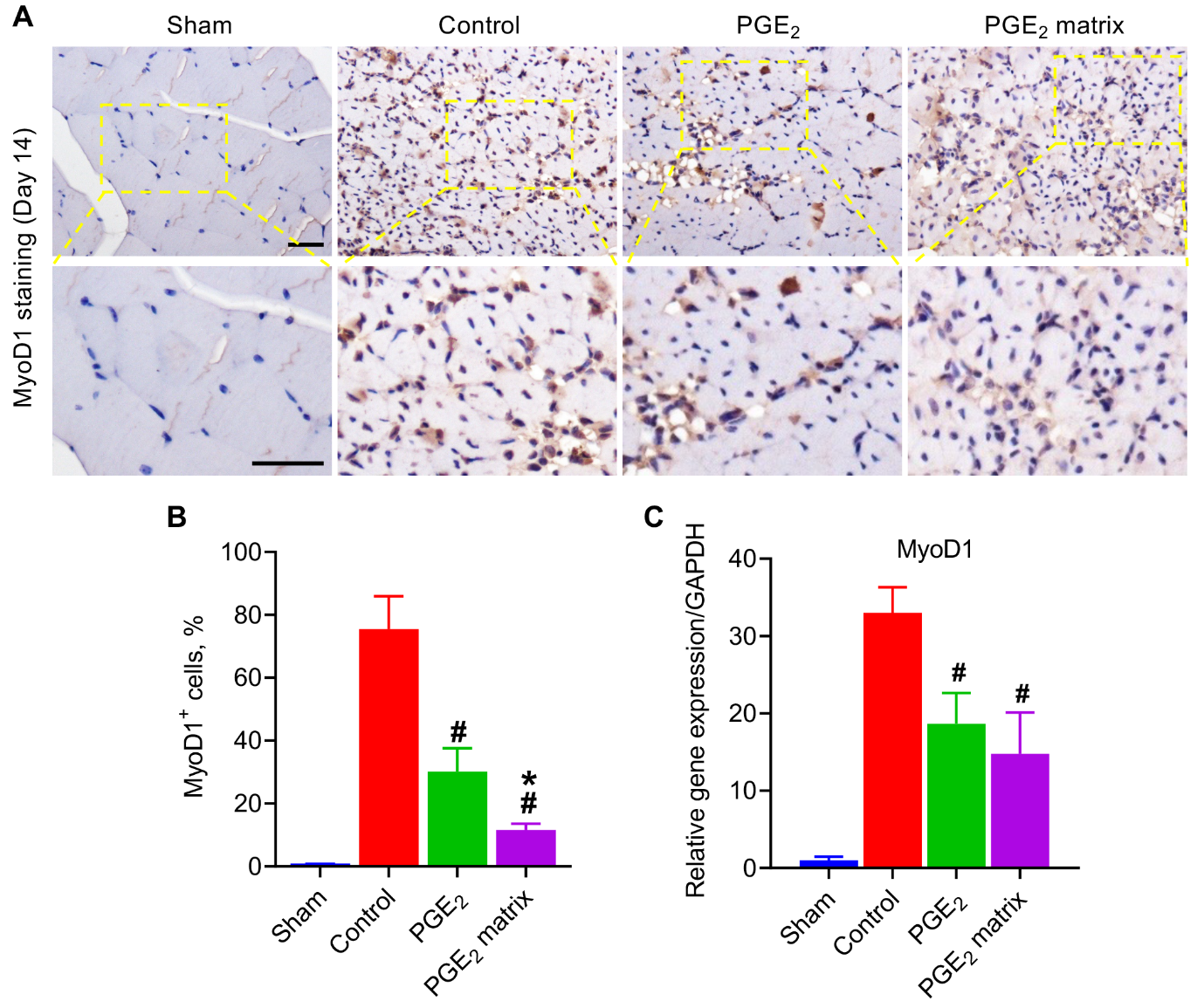


**Supplementary Fig. 3 The time course of MyoD1 in ischemic muscle tissues treated with the PGE_2_ matrix. (A)** Representative images of immunohistochemical staining of MyoD1in muscle tissue from different groups on day 14. Scale bar, 100 *μ*m. **(B)** Quantification of MyoD1 immunostaining in injured muscles. **(C)** Gene expression of MyoD1 in muscle tissue of different groups on day 14. Data are expressed as the mean ± SD. *n* = 5, **P* < 0.05 versus PGE_2_ group; ^#^*P* < 0.05 versus control group. Hindlimb ischemia mice injected with collagen as a control group.


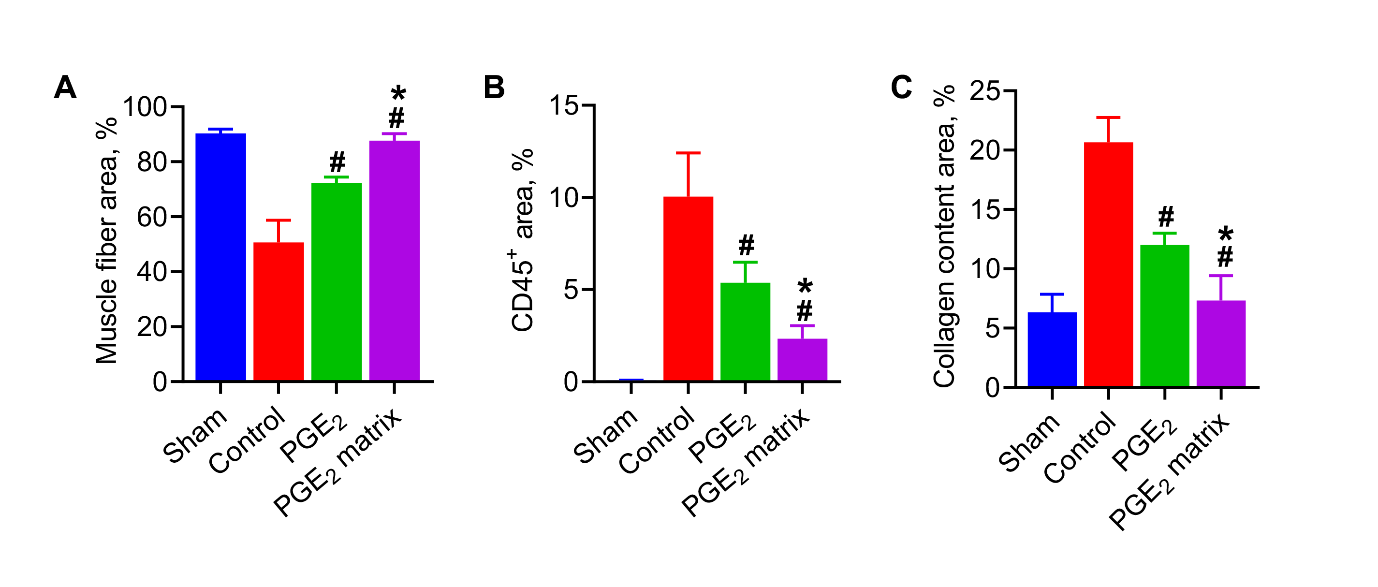


**Supplementary Fig. 4 PGE_2_ matrix ameliorated tissue injury in ischemic hindlimbs.** (**A**) (**A**) Measurement of the muscle fiber area of damaged muscle tissues. **(B)** Quantification of CD45 immunostaining in injured muscles. **(C)** Quantification of the area of fibrosis. Data are expressed as the mean ± SD. *n* = 5, **P* < 0.05 versus the PGE_2_ group; ^#^*P* < 0.05 versus the control group. Hindlimb ischemia mice injected with collagen as a control group.


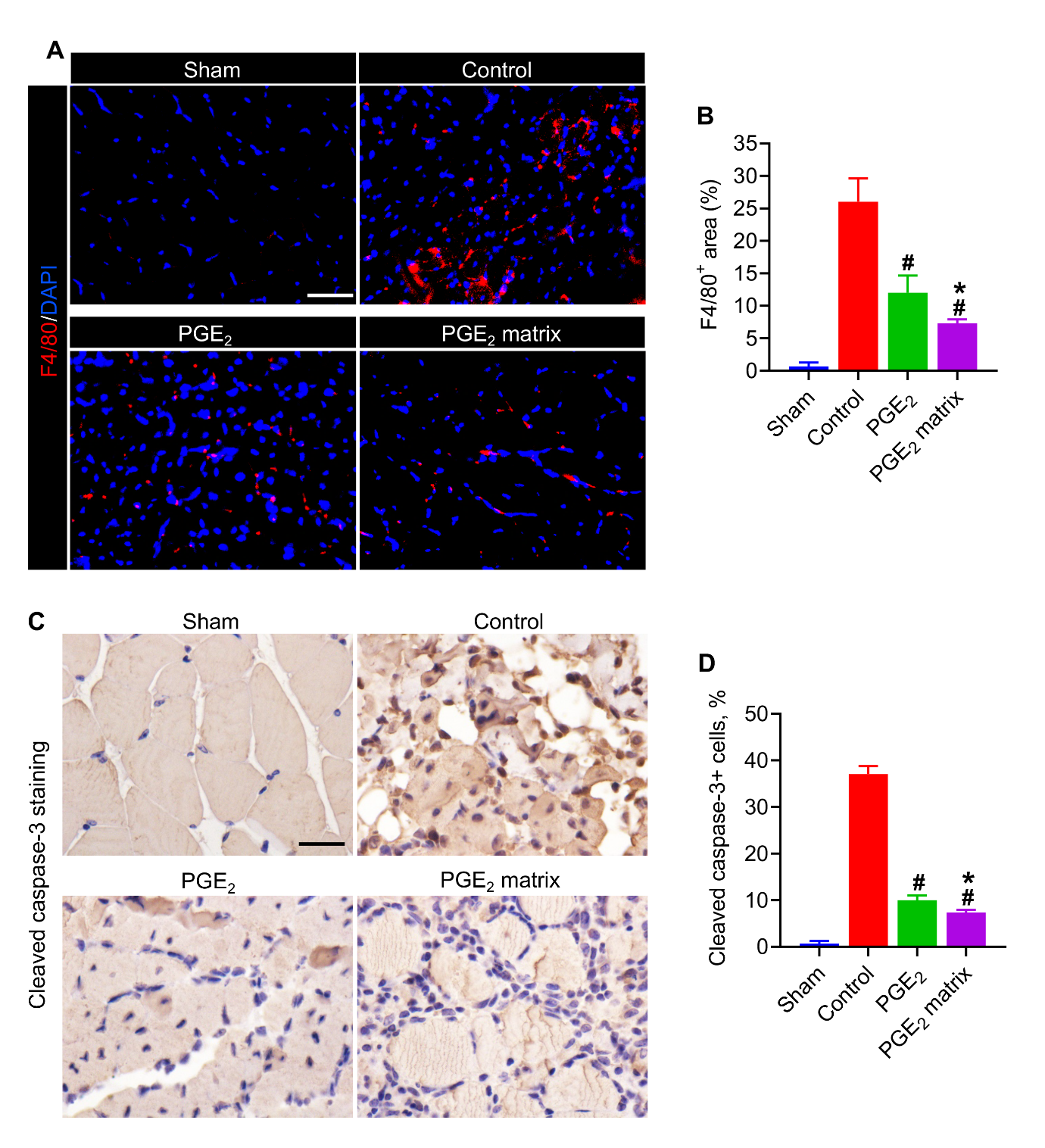


**Supplementary Fig. 5 PGE_2_ matrix inhibited inflammatory responses and prevented apoptosis *in vivo.* (A)** Representative immunofluorescent images of F4/80 expression in muscle tissue from different groups at day14. Red: F4/80; Blue: DAPI. Scale bar, 100 μm. **(B)** Quantification of F4/80 immunostaining in injured muscles. **(C)** Immunohistochemical staining of cleaved caspase-3 on day14 after hindlimb ischemia. Scale bar, 50 μm. **(D)** Quantification of cleaved caspase-3 immunostaining in injured muscles. Data are expressed as the mean ± SD. *n* = 5, **P* < 0.05 versus PGE_2_ group; ^#^*P* < 0.05 versus control group. Hindlimb ischemia mice injected with collagen as a control group.


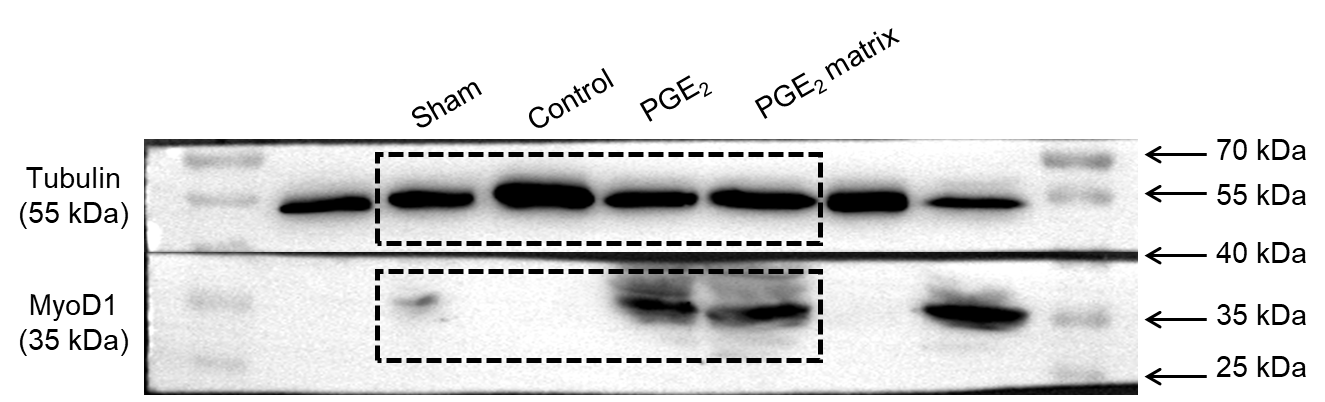


**Supplementary Fig. 6** Images of the uncropped immunoblots shown in Fig. 7D. Boxes indicate cropped regions.
